# Pain management in acute otitis media: a qualitative study exploring GPs’ views and expectations parallel to a trial of an educational intervention

**DOI:** 10.3399/bjgpopen18X101620

**Published:** 2018-10-31

**Authors:** Rick T van Uum, Alies Sjoukes, Roderick P Venekamp, Anne GM Schilder, Esther de Groot, Roger AMJ Damoiseaux, Sibyl Anthierens

**Affiliations:** 1 GP Trainee and PhD Candidate, Julius Center for Health Sciences and Primary Care, University Medical Centre Utrecht, Utrecht, The Netherlands; 2 GP Trainee, Julius Center for Health Sciences and Primary Care, University Medical Centre Utrecht, Utrecht, The Netherlands; 3 GP and Assistant Professor, Julius Center for Health Sciences and Primary Care, University Medical Centre Utrecht, Utrecht, The Netherlands; 4 Ear, Nose and Throat Surgeon, Professor of Paediatric Otorhinolaryngology, and National Institute for Health Research Professor, evidENT Ear Institute, University College London, London, UK; 5 Ear, Nose and Throat Surgeon, Professor of Paediatric Otorhinolaryngology, and National Institute for Health Research Professor, Julius Center for Health Sciences and Primary Care, University Medical Centre Utrecht, Utrecht, The Netherlands; 6 Assistant Professor of Education and Evidence-based Medicine in General Practice, Julius Center for Health Sciences and Primary Care, University Medical Centre Utrecht, Utrecht, The Netherlands; 7 GP and Professor of Primary Care, Julius Center for Health Sciences and Primary Care, University Medical Centre Utrecht, Utrecht, The Netherlands; 8 Primary Health Care Sociologist and Postdoctoral Researcher, Department of Primary Care and Interdisciplinary Care, Antwerp University, Wilrijk, Belgium

**Keywords:** acute otitis media, otitis media, primary health care, pain management, qualitative research

## Abstract

**Background:**

Optimal pain management is accepted as the cornerstone of acute otitis media (AOM) management, but analgesics are neither prescribed routinely nor explicitly recommended in day-to-day practice.

**Aim:**

To explore GPs views on and expectations regarding pain management in children with AOM, and how a multifaceted educational intervention aimed at optimising pain management shapes these perceptions.

**Design & setting:**

Qualitative study conducted alongside a cluster randomised controlled trial (cRCT), the PIMPOM study, in the Netherlands.

**Method:**

Twelve GPs were purposefully sampled from primary care centres allocated to the intervention group and were interviewed, using semi-structured, audio-recorded interviews. The intervention comprised a blended GP educational programme (internet-based and face-to-face training) aimed at discussing pain management proactively with parents using a parent information leaflet, and prescribing paracetamol and ibuprofen according to current guidelines. Interviews were transcribed verbatim and analysed thematically by a multidisciplinary team.

**Results:**

GPs considered AOM a very painful condition. Initially, GPs felt unable to offer adequate treatment for AOM-related ear pain. The intervention provided tools, such as knowledge, communication skills, and an information leaflet, which reduced their feelings of helplessness and empowered them to manage childhood AOM more adequately. GPs indicated that the intervention led to a shift in focus from treating the infection with antibiotics to treating symptoms with analgesics. There was a general lack of knowledge about the possibility of prescribing ibuprofen to children. GPs expressed mixed views on prescribing this drug to children.

**Conclusion:**

A primary-care based multifaceted educational intervention aimed at optimising pain management in childhood AOM offered GPs tools to optimise management of this condition and changed GPs perceptions, namely from treating the infection with antibiotics to treating symptoms.

## How this fits in

Optimal pain management is accepted as the cornerstone of AOM management, but analgesics are neither prescribed routinely nor explicitly recommended in day-to-day practice. This qualitative study shows that GPs feel unable to offer adequate treatment for AOM-related ear pain. An educational intervention empowered them to improve their AOM management, and focus more on treating symptoms with analgesics. Addressing these topics aids further optimisation of childhood AOM management in primary care.

## Introduction

Ear pain is a key symptom of childhood AOM^1^ and is central to children’s and parents’ experience of the illness.^2,3^ Clinical practice guidelines therefore emphasise the importance of analgesics in childhood AOM management.^1,4^ Current evidence, however, indicates that these recommendations are not routinely adopted in day-to-day practice since analgesics are often neither prescribed routinely nor explicitly recommended in this condition.^5,6,7^

Insights into the underlying mechanisms of this routine practice, including GPs’ perceptions on and experiences with analgesia in childhood AOM, are currently lacking. It is known that parents feel unhappy if symptoms of concern, such as ear pain or fever, are not properly addressed.^8^ Yet, parents often consider analgesia as stand-alone treatment insufficient to manage AOM adequately and feel this option is inferior to antibiotic treatment.^7,9^ GPs, in turn, often resort to antibiotics in order to actually do something in situations of diagnostic uncertainty or perceived parental pressure.^8^ This practice in turn reinforces parents in their perception that antibiotics are needed when their child suffers from AOM.^9^

To improve childhood AOM management, a primary care-based multifaceted educational intervention was developed aimed at optimising pain management, and its clinical and cost-effectiveness was assessed in a cRCT.^10^ Mechanisms by which interventions may or may not work are generally hard to identify without examining the underlying processes,^11^ processes that can be unravelled by qualitative research. Such research may also provide important information about the use of the multifaceted intervention in daily practice, and facilitators and barriers of its use; and insight into whether or not trial outcomes can be reproduced in another context.^11,12,13^

This study aims to explore GPs’ views on and expectations regarding pain management in children with AOM, and how a multifaceted educational intervention aimed at optimising pain management shapes these perceptions.

## Method

### Study design

For this qualitative study, semi-structured interviews were conducted with GPs of practices allocated to the intervention group of the primary care-based cRCT. The rationale and design of this trial have been described in detail elsewhere.^10^ In short, 37 Dutch primary care centres (PCCs) were randomly allocated either to a primary care-based, multifaceted educational intervention (intervention group) or to ‘care as usual’ (the control group). The intervention comprised a blended GP educational programme (online training module and face-to-face meeting with the trial’s study physician) aimed at helping GPs learn to discuss pain management proactively with parents using a parent information leaflet, and asked GPs to prescribe both paracetamol and ibuprofen according to current guidelines (despite both drugs being available over-the-counter [OTC] in the Netherlands). The online training module combines various elements, including case-based learning; information on up-to-date clinical guidelines; fragments of interviews between GPs and parents of children with AOM; multiple-choice questions with immediate feedback, as well as open questions; and video demonstrations of consultation techniques. In the face-to-face meeting, the study physician discussed potential barriers for GPs to prescribing analgesics in general, and ibuprofen in particular. The parent information leaflet contains background information on the use of paracetamol and ibuprofen, explains the importance of adequate pain management, and includes age- and weight-adjusted dosing schemes.

### Setting and participants

The GPs were purposefully sampled from all PCCs randomly allocated to the intervention group, taking into account factors such as GP’s age, experience, and PCC characteristics. It was expected that approximately 10–20 semi-structured interviews would need to be conducted to reach saturation.^9,14^

### Data collection and analysis

A study physician interviewed GPs after obtaining written informed consent. The interviewer used a topic list (available from the authors on request) which was based on a review of relevant literature and expert input from all members of the multidisciplinary research team. All interviews were digitally recorded and transcribed verbatim by an independent agency.

Data were collected and analysed in an iterative process using thematic analysis.^15,16^ Thematic analysis was conducted using open and axial coding, making constant comparisons between responders. Two researchers read the first three interview transcripts thoroughly, coded them, and categorised them using Quirkos (version 1.4.1). Codes and emerging themes were discussed in a panel meeting of four researchers. At this time, modifications were made to the topic list to allow for more in-depth exploration. Three researchers analysed the remaining interviews. The coding was reviewed by all team members on a regular basis to reach consensus about the interpretations and to increase trustworthiness.^17^ All research team members reviewed the interpretation of the main themes from the interim analysis during a consensus meeting. Data were collected until saturation was reached. In the final analysis, all researchers revised the established themes based on the data collected since the panel meeting.

## Results

In total, one researcher performed 12 interviews (mean duration 30 minutes) between June 2016 and May 2017, at which point saturation was reached. Table 1 provides an overview of the characteristics of participating GPs. The results that emerged from the interviews could be summarised in three main themes: shift from treating infection to treating symptoms; empowerment of GPs through the intervention; and balancing potential beneficial and harmful effects of prescribing ibuprofen to children.

**Table 1.** Characteristics of participants

Participant number	Sex	Age, years	Qualification	GP experience, years	Working days, (days/week)	Practice characteristics		
						**Young versus old^a^**	**Predominantly Dutch versus multicultural**	**Rural versus urban**
1	F	44	MD	17	4.0	Young	Predominantly Dutch	Urban
2	M	37	MD	8	4.0	Young	Predominantly Dutch	Urban
3	F	59	MD	32	4.0	Young	Predominantly Dutch	Rural
4	M	52	MD	13	5.0	Young	Predominantly Dutch	Urban
5	F	41	MD	9	3.0	Young	Predominantly Dutch	Urban
6	M	33	MD	5	4.5	Old	Predominantly Dutch	Semi-rural
7	M	60	MD	23	4.0	Old	Predominantly Dutch	Semi-rural
8	M	62	MD	36	4.0	Old	Predominantly Dutch	Rural
9	F	32	MD	4	3.0	Old	Predominantly Dutch	Rural
10	F	52	MD	25	4.0	Old	Predominantly Dutch	Rural
11	F	59	MD, PhD	28	3.0	Old	Predominantly Dutch	Urban
12	M	62	MD	32	4.0	Young	Predominantly Dutch	Urban

^a^A young practice is defined as over 10.9% of patients in practice being aged <10 years. Cut-off value is based on national figures; in the Netherlands 10.9% of the population is aged <10 years.^23^

F = female. M = male. MD = medical doctor. PhD = doctor of philosophy.

### Treating symptoms instead of infection

All GPs stated that ear pain is a frequent reason for parents of children with AOM to consult a doctor. GPs considered AOM to be a very painful condition. A few GPs could even remember their own childhood AOM episode since it had been so painful. Others expressed that their own children had suffered from AOM-related ear pain. One commented:

*'Well, an earache is incredibly painful. If you have ever had one yourself, you know enough. You’re ready to bang your head against the wall, that’s how much it hurts.'* (P9)

However, one GP realised the severity of the ear pain caused by AOM through the internet-based training and felt that she had underestimated this condition thus far. She stated that clinicians are obligated to treat (ear) pain adequately. This was reflected by other GPs, who stated that the knowledge (whether pre-existing or kindled by the intervention) that childhood AOM is generally accompanied by considerable ear pain motivates them to provide adequate pain management; they felt that it warrants further action by the GP:

*'Most important to me is that they take that earache seriously. A child with an earache, it just really hurts, so they* [parents] *have to give painkillers.'* (P8)

GPs indicated that, prior to participating in the training, they mostly focused on whether or not to prescribe antibiotics, with analgesia considered less important.

The intervention provided new knowledge on analgesia and, as a result, GPs expressed that they made symptom relief central to their consultation by proactively discussing pain management with parents.

GPs indicated that the intervention led to a change in AOM pain management. GPs who tended to advise parents to administer paracetamol three times daily in a sufficient dosage did now prescribe ibuprofen as add-on therapy. GPs who irregularly advised on analgesics, or who took parents' response that they had already administered paracetamol to their child for granted, now tended to better explore the amount and dosage of paracetamol administration, and — where appropriate — advised parents to administer a higher dosage of paracetamol, or explicitly stressed the importance of administering paracetamol on a more regular basis:

*'So, I started to shift, I think, from treating the infection specifically and pointing that out, to the pain aspect, which tends to be far more important to the parents.'* (P5)

*' ... but then I did actually state much more clearly that pain relief was important, that antibiotics weren’t useful here while analgesics were, the importance of a consistent pain medication level and, well, as a rule you can take paracetamol three times a day, and then there’s still ibuprofen on top of that.'* (P1)

Several GPs did express that they prescribed fewer antibiotics to children with AOM due to the increased focus on pain management. Not all GPs shared this opinion though; some felt there was no difference in their number of antibiotic prescriptions for AOM, whereas others were unsure whether antibiotic prescribing changed. A subset of GPs indicated that the intervention led to fewer re-consultations and subsequently fewer antibiotic prescriptions. The interviewer asked if they had noticed that they had started to prescribe antibiotics more often, less often, or the same amount:

*'I’m pretty reticent to do so anyway, I think. So if you ask me, not much has changed.'* (P9)

*'But what I do know is that, of the children I signed up for the study, I didn’t see any of them again. So, they were never given antibiotics, in any case.'* (P3)

### Empowerment of GPs through the intervention

Prior to the intervention, several GPs struggled with the idea of not being able to offer adequate treatment for AOM-related ear pain. Some parents had already provided paracetamol to their children before consulting the GP, and GPs therefore felt they could not offer these parents a solution for their child’s symptoms and did send those children home without additional analgesics. In the end, several of these children re-consulted their GP because of persisting symptoms, and were then prescribed antibiotics, even though the GP felt a clear indication was lacking.

The new knowledge on analgesia offered by the intervention might have changed their attitudes towards the management of these patients. It diminished GPs’ feeling of helplessness and increased their self-efficacy in managing these patients. GPs particularly valued the positive message that they could actually do something, and considered the intervention easily implementable:

*'It is really annoying to have nothing to offer. Well, you do have something to offer: a sympathetic ear, an explanation, empathy. But sometimes it’s not enough and people want to have something. And then it’s nice to have something to give.'* (P4)

*'Yet I used to look only at the infection, and say: "Well, he does have an infection but it’ll pass, just give him some paracetamol." Whereas now, you’re drawing up an actual pain medication schedule. Which as a doctor, gives me more of a sense of doing something, as well as providing the parents with tools to help their child in pain, the one with persistent symptoms, to treat them.'* (P5)

Most GPs indicated they extended their history taking by asking parents whether they had provided analgesics to their child and, if so, which type, how frequent, and at which dosage. This communication technique, exploration, was specifically addressed in the internet-based training to optimise pain management. One GP commented:

*'These days, I do, in fact, ask people much more frequently how they fight it. Your study really was a wake-up call for me, so to speak. That idea of asking: what are they doing now, how often are they giving it, and what, specifically, do they give. And are they administering that thing preventively or not. So in that regard, I do find the study appealing, because you take a different approach than in the past.'* (P3)

Furthermore, GPs were very positive about the parent information leaflet. All GPs stated that they used the leaflet specifically to illustrate the specific dose per weight recommendations of paracetamol and ibuprofen, but not to inform parents about AOM or analgesics in general. Several GPs also used the leaflet for children who did not participate in the trial or who consulted them because of other painful conditions. Some even used the leaflet, or copies thereof, during their out-of-hours service:

*'Well, I even took it with me to the after-hours clinic; I put it in my bag, and then I just grab it, indicate the level, and then calculate* [the dose]*. People typically ask me for written instructions on how much they’re supposed to take.'* (P1)

*'Yes. It always adds a certain panache, of course, when a doctor prescribes something. I felt even more — because I did use that card you gave me with that dosage — even more like "This doctor knows exactly how much it has to be, because she has worked it out very precisely." That would provide motivation, whether it was prescribed or not.'* (P10)

The intervention asked GPs to prescribe, rather than recommend, analgesics according to current guidelines. In the Netherlands, both paracetamol and ibuprofen are available OTC and therefore a prescription is not formally required. In general, GPs valued prescribing analgesics (in particular ibuprofen), as they felt that a prescription increased the trustworthiness of drugs and might lead to better compliance, and improved medical documentation for themselves and colleagues:

*'Why I make that distinction? Good question. I guess also because it’s a little more familiar and the parents feel that a prescription is more effective, I think, than me just telling them to go to a drugstore and pick up some OTC syrup.'* (P10)

In everyday practice, however, GPs usually decided to provide a prescription for ibuprofen, but not for paracetamol, since they assumed most parents are more familiar with it and would have paracetamol at home. Some GPs were unfamiliar with the OTC availability of Nurofen (liquid ibuprofen) and therefore chose to specifically prescribe this, yet other GPs thought a prescription might provide parents with explicit instructions on ibuprofen usage, assuming parents were unfamiliar with administering ibuprofen to their children, in addition to the information provided in the booklet.

### Balancing potential beneficial and harmful effects of prescribing ibuprofen to children

None of the GPs were familiar with prescribing ibuprofen to children suffering from pain. There was a general lack of knowledge about the possibility of prescribing ibuprofen at a young age (≥1 year). Ibuprofen for children was regarded as a neglected subject in Dutch primary care and GP training, and some GPs felt they had insufficient knowledge and experience to safely prescribe ibuprofen in children prior to the intervention. Once presented with this information, some GPs indicated they were inclined to prescribe ibuprofen and were eager to have a tangible additional management option of potential benefit for their patient:

*' ... and indeed, the addition of ibuprofen.* […] *Because I didn’t know that it was appropriate for children this young.'* (P1)

*'That’s really the most important message, I think, to keep in mind: that there’s nothing wrong with three days of ibuprofen use.'* (P4)

*'And the thing I like is,* [it shows] *that you have certain preconceived notions. My own understanding, for sure, was that ibuprofen was unacceptable for children. And it was entirely unfounded. It’s not like that idea was promoted in medical school, that you shouldn’t* [use it] *with children. In fact, it’s something that was never really touched upon.'* (P6)

On the other hand, some GPs already knew ibuprofen could be administered to young children, but felt reluctant to prescribe this drug since none of their colleagues did so. Other GPs felt reluctant since non-steroidal anti-inflammatory drugs such as ibuprofen have received negative media attention in recent years. Over the past decade, awareness of ibuprofen-related adverse effects has increased and clinicians are explicitly recommended to be cautious when prescribing ibuprofen to older people. Children are considered even more susceptible and vulnerable to adverse effects than older people, and GPs therefore refrain from prescribing ibuprofen.

These GPs, however, now consider prescribing ibuprofen if the child suffers from pain and has already received an optimal dosage of paracetamol. It seems the need for better pain management overcomes their hesitation to prescribe ibuprofen:

*'Well, in any case, what I took away is what I learned from the introductory videos, about the low barriers to use ibuprofen in children.'* (P7)

## Discussion

### Summary

This qualitative study revealed that, while GPs considered AOM to be a very painful condition, they initially felt unable to offer adequate treatment for AOM-related ear pain. GPs indicated that a multifaceted educational intervention aimed at optimising pain management in childhood AOM offered them tools to improve management of this condition. Particular components of the intervention such as knowledge (for example, on ibuprofen as a treatment option for AOM), communication skills, and dosing schedules in the parent information leaflet reduced GPs’ feelings of helplessness and empowered them to manage childhood AOM more adequately by increasing their self-efficacy. The GPs also expressed that the intervention led to a paradigm shift, from treating the infection with antibiotics to treating symptoms with analgesics.

### Comparison with existing literature

Previous studies indicated that multifaceted interventions or active continued medical education are able to change clinicians’ behaviour,^18,19^ but failed to identify mechanisms by which these interventions may have worked. This qualitative study provided both insights into GPs’ perceptions on and experiences with pain management in children with AOM, and unravelled underlying processes by which a multifaceted educational intervention to optimise pain management shaped their perceptions on AOM management.

Typically, the process of changing clinicians’ behaviour is complex, and consists of multiple stages.^20,21^ Not only do clinicians need to be aware of the value of potential change (cognition), they also have to form an image of alternatives to current practice, learn what they need to initiate change, be convinced of the necessity to change (motivation), and subsequently implement the desired changes into practice. In addition, social influence, organisational aspects, and resources have an influential role.^21^ This intervention addresses multiple stages, thereby enhancing the potential to change: it raises awareness among GPs about the frequency and painfulness of AOM-related ear pain; provides explicit information about the impact of the condition on both children and parents, and on the use of analgesia; and offers tools to discuss pain management proactively with parents within the consultation, using effective communication techniques and a parent information leaflet. GPs gained more knowledge and felt more empowered through the intervention. The general belief that 'withholding antibiotics equals doing nothing' was replaced by the feeling of being able to support the patient, through optimal analgesia. Eventually, through more empowered GPs, the intrinsic motivation to treat AOM in an optimal way may increase. Since clinicians’ attitudes are central to their prescribing behaviour,^22^ empowerment may further contribute to a change in behaviour. In the end, a more optimal pain management may result in a more fruitful consult, as parents’ concerns might be more properly addressed.^8^

### Strengths and limitations

As part of the intervention, GPs were asked to prescribe paracetamol and ibuprofen, despite both drugs being available OTC. Although GPs generally valued this element, one could argue that this strategy may lead to a sustained dependency of parents of children experiencing AOM symptoms on a GP prescription and might subsequently lead to an increase in healthcare utilisation and associated costs. Further qualitative research with parents and quantitative research on the actual number of prescriptions filed in the trial are needed to address this important issue.

Based on current findings, GPs seem enthusiastic about the multifaceted educational intervention, and value its impact on their management of childhood AOM. Some of the participating GPs did not enrol any children to the trial but did implement some of the components of the intervention in everyday childhood AOM management or for other conditions such as sore throat, suggesting its reach may extend beyond research and into daily practice.

The trustworthiness of these findings was established through researcher triangulation, as coding and analysis was performed by multiple researchers. In the multidisciplinary research team, the different frames of reference of a primary healthcare sociologist; an ear, nose, and throat surgeon; an educational specialist; and academic GPs contributed to a broad scope of data interpretation. In terms of reflexivity, trustworthiness was enhanced through a multidisciplinary approach in all phases of analysis, to prevent distortion of data interpretation as a result of one researcher both performing the interviews and introducing the intervention. Furthermore, GPs’ perceptions were explored prior to trial completion, which allowed analysis of these concepts in a more neutral way than would have been possible with knowledge of the trial results. To avoid an overly optimistic attitude towards the intervention, GPs were actively encouraged to provide negative feedback on the intervention at all times, and were also questioned on general topics concerning analgesia, as well as interaction with parents. Finally, since the studied elements of behaviour — for example, awareness, beliefs, and prescribing behaviour — are relevant to all GPs, regardless of doctor and PCC characteristics, the authors believe that these results are widely transferrable.

### Implications for research and practice

In conclusion, this qualitative study illustrates that although GPs considered AOM to be a painful condition, they initially felt unable to offer adequate treatment for AOM-related ear pain. GPs expressed that a multifaceted educational intervention aimed at optimising pain management in childhood AOM empowered them to manage this condition more adequately and changed their perceptions on AOM management, from treating the infection with antibiotics to treating symptoms.
